# Antioxidant activity at the molecular level: exploring ways of action and computational tools to investigate them

**DOI:** 10.1039/d5sc05463j

**Published:** 2025-10-09

**Authors:** Annia Galano

**Affiliations:** a Departamento de Química, Universidad Autónoma Metropolitana-Iztapalapa Av. Ferrocarril San Rafael Atlixco 186, Col. Leyes de Reforma 1 A Sección, Alcaldía Iztapalapa Mexico City 09310 Mexico agal@xanum.uam.mx

## Abstract

Despite their apparent simplicity, antioxidants are involved in numerous and complex processes. Several key aspects of antioxidant chemistry are covered in this review. (I) Their ways of action, which include scavenging free radicals; inhibition of ˙OH production *via* Fenton-like reactions by chelating redox metals; the repair of oxidatively damaged biomolecules; and modulation of the antioxidant/oxidant enzymatic system. (II) The main mechanisms involved in those ways of action, such as formal hydrogen atom transfer (*f*-HAT), single electron transfer (SET), sequential proton lost electron transfer (SPLET), coupled-deprotonation–chelation mechanism (CDCM), oxidant-enzyme inhibition, and antioxidant-enzyme activation. (III) Computational tools aiming to explore antioxidant activity (AOX). They are roughly grouped into four categories, depending on the used strategy (calculated properties): reactivity descriptors, thermochemistry, kinetics and ligand–receptor interactions. The approaches used to estimate them include calculations based on activity–structure relationships, quantum mechanical calculations, and molecular docking. The limitations and advantages of using these strategies and approaches are discussed, as well as some key points related to mimicking the associated chemical reactions (*e.g.* the importance of solvent polarity, pH, and diffusion). (IV) Some future research directions in the field, like the computational design of new (more efficient) antioxidants, and the emerging role of machine learning (ML) and artificial intelligence (AI) as efficient strategies to address AOX, can contribute to gaining a more complete picture about the complex chemical behavior usually involved in the health benefits offered by antioxidants.

## Abstract 2

Antioxidant protection involves complex chemistry, related to their versatile ways of action. Identifying suitable candidates for this purpose, requires more than one criterion; otherwise, predictions should be cautious and specific.

## Graphical abstract

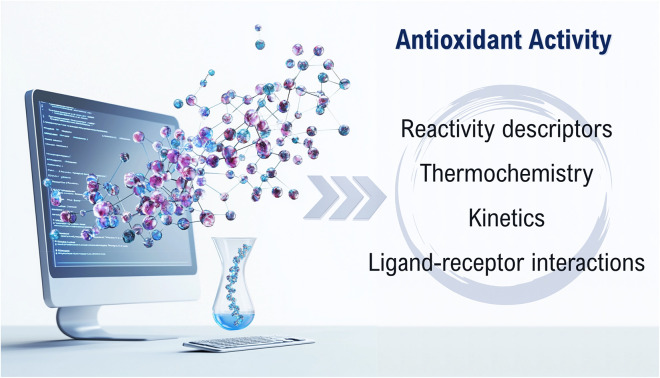

## 1. Introduction

**Fig. 1:**
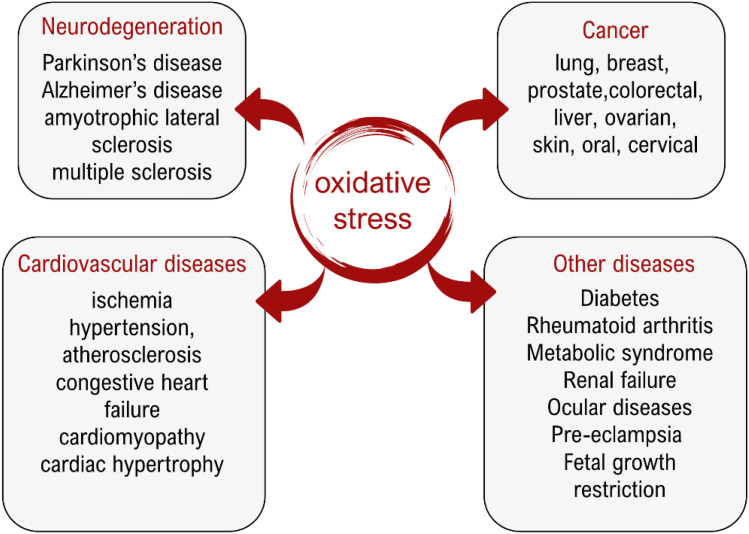
Some OS-related diseases.

Oxidative stress (OS) is a form of chemical stress in biological systems that occurs when oxidants, primarily free radicals (FRs), exceed healthy levels. It arises as a consequence of antioxidant defenses failing to counteract the overproduction of, or overexposure to, highly reactive FR species. The damage caused by OS to key biomolecules promotes aging and a variety of chronic and degenerative diseases (Fig. 1).^1–5^ Thus, antioxidants have emerged as adjuvants in the treatment of several OS-related health disorders.^6–10^

Although the word “antioxidant” is commonly used, its definition has been unclear. Haliwell and Gutteridge proposed that an antioxidant is “Any substance that, when present at low concentrations compared with those of an oxidizable substrate, significantly delays or prevents oxidation of that substrate”.^11^ Here, the term oxidizable substrate refers to bioorganic molecules that are found *in vivo*. More recently, after considering the large variety of processes through which substances can decrease OS, they simplified the definition to: “any substance that delays, prevents, or removes oxidative damage to a target molecule”.^12^ The latter definition is the one used in this review since it covers the diverse ways of action in which antioxidants may be involved. Free radical scavenging (FRS) contributes to both delaying and preventing OS; inhibiting ˙OH production (IOP), when it involves Fenton-like reactions, and promoting enzymatic protection (PEP) also contribute to OS prevention; and repairing damaged biomolecules (RDB) helps removing OS.

In addition, those ways of action arise from various mechanisms, including both chemical and enzymatic pathways.^13^ The most widely studied, when using computational tools, is the antioxidants' FRS capability. This is frequently done based on intrinsic reactivity descriptors, such as bond dissociation energies (BDEs) or ionization energies (IEs). However, as useful as it is, this approach is not general enough to assess antioxidant activity (AOX). Because FRS is not the only way in which antioxidants can protect against OS,^14–16^ and there are other factors such as the nature of the radical counterpart and reaction rates that influence AOX.

This review is focused on discussing the various processes that contribute to the protective effects of antioxidants and the computational tools that can be used to investigate them. Considering all the ways of action involved in AOX is essential to develop a complete understanding of how they exert their health benefits and to design more efficacious antioxidants.

## 2. Ways of action and mechanisms involved in antioxidant activity

This section briefly summarizes different ways through which antioxidants can act as protectors against OS, since the main purpose of this review is to discuss computational approaches meant to investigate AOX. There are other ways to counteract OS that, although not reviewed here, are important to mention. Ferroptosis, first described by Dixon *et al.* in 2012,^17^ was defined by these authors as “a unique iron-dependent form of nonapoptotic cell death”. Its activation is related to destruction of cancer cells and its inhibition to protection against neurodegeneration. It has also been found that radical-trapping antioxidants inhibit lipid peroxidation and cell death associated with ferroptosis by a mechanism that involves radical addition to the ligand backbone. Several molecular systems capable of exerting this kind of protection have been identified, including diarylamines and their nitroxides,^18^ bazedoxifene,^19^ naphthoquinones,^20^ thiosemicarbazones metal complexes,^21,22^ organoboranes,^23^ and hydroxyoestradiol derivatives.^24^

### 2.1. Free radical scavenging (FRS)

In this context, it seems worthwhile to make some comments regarding free radicals, especially those considered when computationally modelling AOX, *via* FRS. As previously pointed out by Koppenol and Hider,^25^ the term “reactive oxygen species” (ROS) may be misleading. It includes a wide variety of chemical species, such as O_2_˙^−^, ^1^Δ_*g*_ O_2_, H_2_O_2_, ˙OH, alkoxyl, and peroxyl radicals. The superoxide radical anion is not an oxidant; in contrast, it is a strong reductant. Singlet oxygen is not produced in animals, and water molecules very quickly quench it. The reactivity of H_2_O_2_ is very low, and it is mainly scavenged enzymatically. In contrast, the hydroxyl radical is so highly reactive that it would react with almost any molecule at diffusion-limited rates. Thus, using it as a model free radical in calculations might lead to the identification of a candidate as a promising antioxidant, when it is not. What can then be used as an appropriate counterpart for antioxidants when investigating their FRS activity? Peroxyl radicals are well-suited for this purpose. They have moderated reactivity, which allows them to have relatively long half-lives^26^ and reach remote locations. They are formed in living systems, where they damage DNA, lipids, and proteins.^27^ Moreover, they have been proposed as key species that antioxidants can efficiently scavenge to inhibit OS.^28^ Even a “peroxyl radical clock” methodology has been developed to measure the rate constants of the reactions between peroxyl radicals and molecules.^29^

**Scheme 1:**
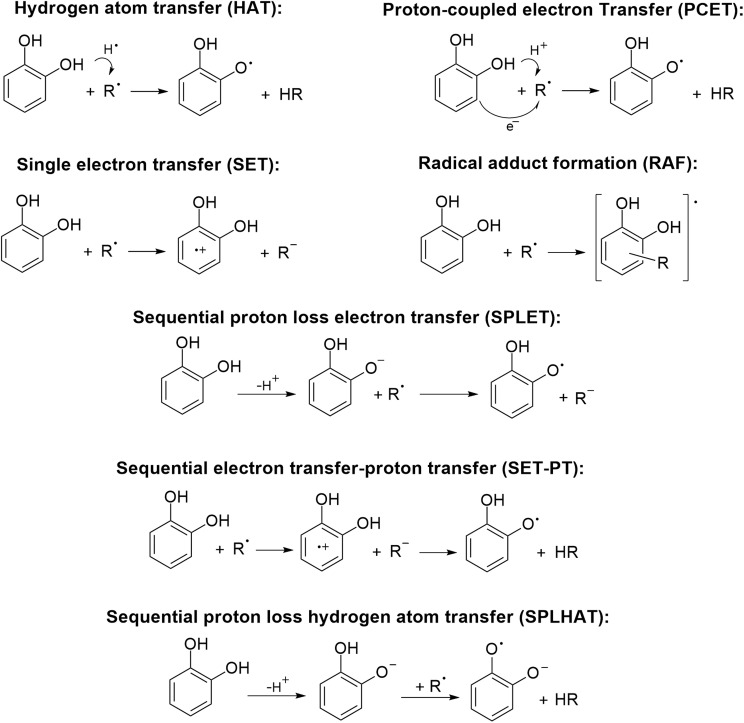
Reaction mechanisms that might contribute to the free radical (R˙) scavenging activity of antioxidants. Catechol is used as a hypothetical example to illustrate the mechanisms.

The FRS way of action is also referred to as primary AOX, type I, or AOX-I. It implies that the antioxidant reacts directly with a free radical (FR), converting it into a less reactive species that no longer poses a hazard to biomolecules. It can occur through various reaction mechanisms.^30^ The most common ones are illustrated in Scheme 1, using catechol as a hypothetical example, although not all these routes significantly contribute to the AOX of this molecule. Other reaction mechanisms have also been proposed for primary antioxidants. Some examples are: the “concerted bimolecular homolytic substitution” for the peroxyl radical scavenging of polysulfides;^31^ the “sequential triple proton loss triple electron transfer”; and the “adduct formation followed by hydrogen atom abstraction” for chalcone derivatives, when reacting with different free radicals.^32^ Examples of molecules for which the mechanisms mentioned in this section (Scheme 1) have been identified to contribute significantly to their AOX are provided in Table 1. It is interesting to note that for curcumin the main mechanism changes depending on the reacting radical and the isomerism.

**Table 1:** Some examples of antioxidants that scavenge free radicals through different mechanisms

	Molecules	Ref.
HAT	Glutathione	33
	Dihydrolipoic acids	34
	Tryptophan	35
PCET	Flavonoids	36
	Curcumin (+˙OOH)	37
	Vitamin E and ubiquinol	38
SET	Enol isomer of curcumin	(ref. 39)^a^
	Galloylated tannins	(ref. 40)^a^
	Planar catechin analogues	(ref. 41)^a^
	Tetrahydrofuran lignans	42
RAF	Carotenoids	43
	Hydroxybenzyl alcohols	(ref. 44)^a^
	Rebamipide	(ref. 45)^a^
	Carnosine	(ref. 46)^a^
SPLET	Curcumin (+DDPH)	(ref. 47)^a^
	Esculetin	48
	Resveratrol	49
	Piceatannol	33
	Trolox	50
SET-PT	Baicalein	51
	Astaxanthin	(ref. 52)^a^
	α-Tocopherol	(ref. 53)^a^
SPLHAT	Anthocyanidins	54
	Chalcone derivatives	32
	Coumarin–chalcone hybrids	55
	Betanidin	56

a Experimental or combined experimental–theoretical studies.

Regarding the mechanisms shown in Scheme 1, HAT, PCET, and SET-PT yield the same products, *i.e.*, the global reaction involves the transfer of an H atom from the antioxidant to the radical. For the sake of simplicity, here, these processes are referred to as formal H atom transfer (*f*-HAT) without distinguishing among them, because such a distinction escapes the purposes of this review. However, it is possible to differentiate between HAT and PCET using quantum chemistry calculations. For example, analyzing the charge on the transferred hydrogen along the reaction coordinate. If the charge is significantly positive, it would indicate that the H is being transferred as a proton, in line with a PCET process. In contrast, if it is ≤0.3, it would suggest a HAT mechanism. Another criterion is the atomic spin density on the atoms involved in the transfer. Spin populations mainly located on the atoms exchanging the H are consistent with HAT. The singly occupied molecular orbital (SOMO) in the TS can also be used to differentiate between these two pathways, under the assumption that the electron and the proton are transferred from different orbitals. A SOMO corresponding to HAT is expected to involve orbitals adjacent to the transition vector, while a SOMO corresponding to PCET involves p orbitals orthogonal to such a vector. It is recommended to use, at least, all these criteria to differentiate between HAT and PCET or the recently proposed strategy that combines intrinsic bond orbital (IBO) formalism, activation strain, and energy decomposition analyses.^57^ The interested reader can find more information about this topic elsewhere.^58–60^

It is also important to mention that SPLET and SET-PT do not yield the same products (Scheme 1), although both routes involve the transfer of one proton and one electron. In the SPLET mechanism, the proton is exchanged with the solvent (water) during the first step, through acid/base equilibria. In the second step, the deprotonated antioxidant transfers an electron to the FR, turning it into an anion. In contrast, in the SET-PT mechanism, both particles are transferred from the antioxidant to the radical, first the electron, then the proton. Thus, in this case, the FR becomes a non-charged closed-shell species (HFR).

### 2.2. Inhibiting ˙OH production (IOP)

As mentioned in the previous section, antioxidants cannot efficiently scavenge hydroxyl radicals because of their high reactivity. The same reason makes them extremely dangerous to the integrity of biomolecules. What antioxidants can do is inhibit the ˙OH production when it occurs *via* Fenton-like reactions:M(rd) + H_2_O_2_ → M(ox) + OH^−^ + OH˙where M(rd) represents the reduced form of a metal, such as Fe(ii) or Cu(i), and M(ox) stands for the oxidized form of the metal, such as Fe(iii) or Cu(ii), respectively. Since these redox metals are mainly present in living organisms as M(ox), inhibiting their reduction would also inhibit the production of ˙OH. That is what molecules, referred to as OH-inactivating ligands (OIL),^61,62^ do. They bind to M(ox), forming complexes that can prevent ˙OH damage in two ways: (i) by inhibiting the metal reduction and, consequently, ˙OH production or (ii) by scavenging the radical immediately after it is produced, since the antioxidant moiety is in close proximity to the ˙OH production site.^63^ This is considered an indirect antioxidant activity.

Some chemical routes might contribute to the chelation processes. Some of them are:

Direct chelation mechanism (DCM):M(ox) + H_2_L → M(ox)–LH_2_

Coupled deprotonation–chelation mechanism (CDCM):M(ox) + H_2_L → M(ox)–LH + H^+^

Coupled double-deprotonation–chelation mechanism (C2DCM):M(ox) + H_2_L → M(ox)–L + 2H^+^

**Scheme 2:**
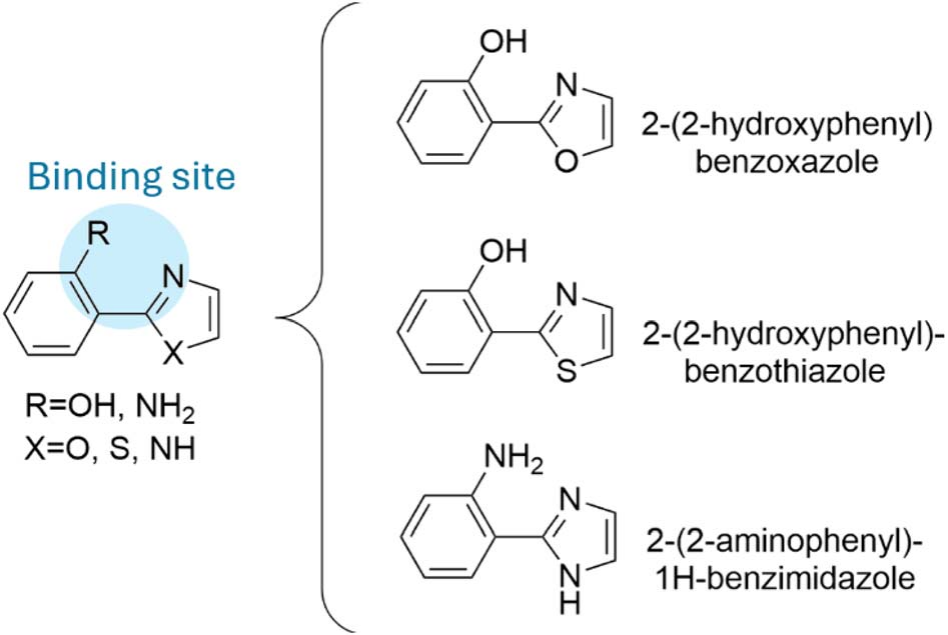
M1 framework and some molecules containing it.

The molecular framework referred to as M1 (Scheme 2) is promising for this purpose, although molecules with other structural features may also be efficient as OIL agents. Some molecules that have the M1 feature are shown in Scheme 2. Based on the performance of this framework as a chelator and on its potential use in the treatment of Alzheimer's disease, several compounds inspired by it have been developed.^64–66^ Other molecules that were proposed to be effective as OIL agents are: capsaicin,^67^ citric acid,^68^ melatonin and some of its metabolites,^69,70^ and ellagic acid.^71^

### 2.3. Repairing damaged biomolecules (RDB)

**Scheme 3:**
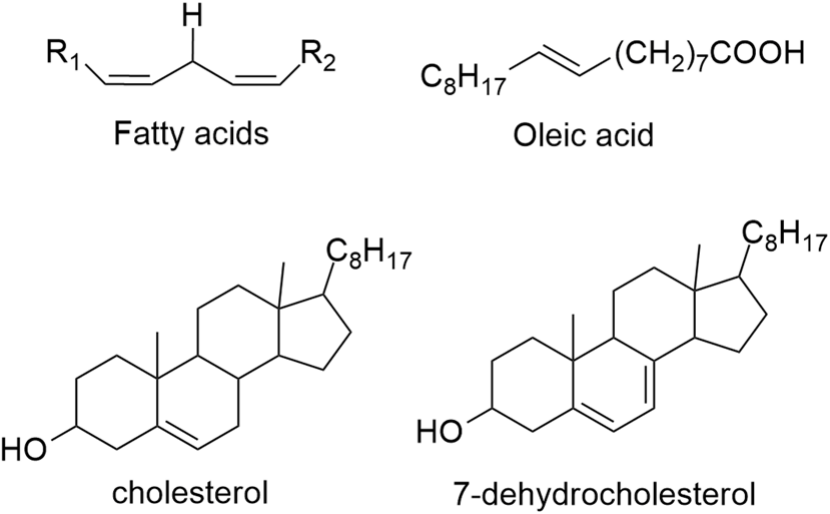
Some lipid substrates susceptible to free radical-induced oxidation.

Another way to counteract the deleterious effects of OS is by repairing the biomolecules damaged by ˙OH and other free radicals. Lipid peroxidation initiates with an H atom transferring from the allylic sites in the lipid to the free radical. Several biological lipidic substrates are highly susceptible to oxidation, including oleic acid, cholesterol, 7-dehydroxycholesterol, polyunsaturated fatty acids (PUFAs), and their esters (Scheme 3).^72^ For linoleic, arachidonic, eicosapentaenoic, and docosahexaenoic acids and their esters, a wide variety of difficult-to-isolate products are produced during oxidation. Such complexity represents a challenge for investigating the full mechanism involved in lipid peroxidation through product distribution. That is why the associated chemistry has been considered a “black box”.^73^ Experimental approaches, based on mass spectrometry combined with HPLC separation, have enabled some progress in elucidating these intricate product profiles and the associated mechanisms. Computational tools can also help achieve that purpose.

OS leading to protein damage is also complex. Several amino acid residues can undergo oxidation initiated by free radicals. The amino acids most susceptible to radical damage are those containing sulfur,^74^ with the main reaction path involving *f*-HAT from the thiol group in cysteine and from the CH_2_ bonded to the S atom in methionine. These amino acids are considered a relevant part of the antioxidant system due to their high reactivity towards FRs.^75–77^ Non-sulfur aliphatic amino + FR reactions involve *f*-HAT processes from their sp^3^ C sites or amino groups. Some that have been identified to contribute to protein oxidation are leucine, isoleucine, serine, and arginine.^78^ For aromatic amino acids, oxidative damage through *f*-HAT is a minor pathway. Instead, their main products correspond to ˙OH-adducts in phenylalanine and tyrosine aromatic groups and in the indole ring of tryptophan.^74,79,80^ However, the oxidation of the latter also produces the corresponding radical cation,^81^ which suggests FR-induced damage *via* SET. Moreover, within proteins, a tyrosine/tryptophan-SET/PCET process has been described to proceed *via* a series of single-step hopping events, involving electron and protons.^81^ This contributes to the complexity of OS damage to proteins.

**Scheme 4:**
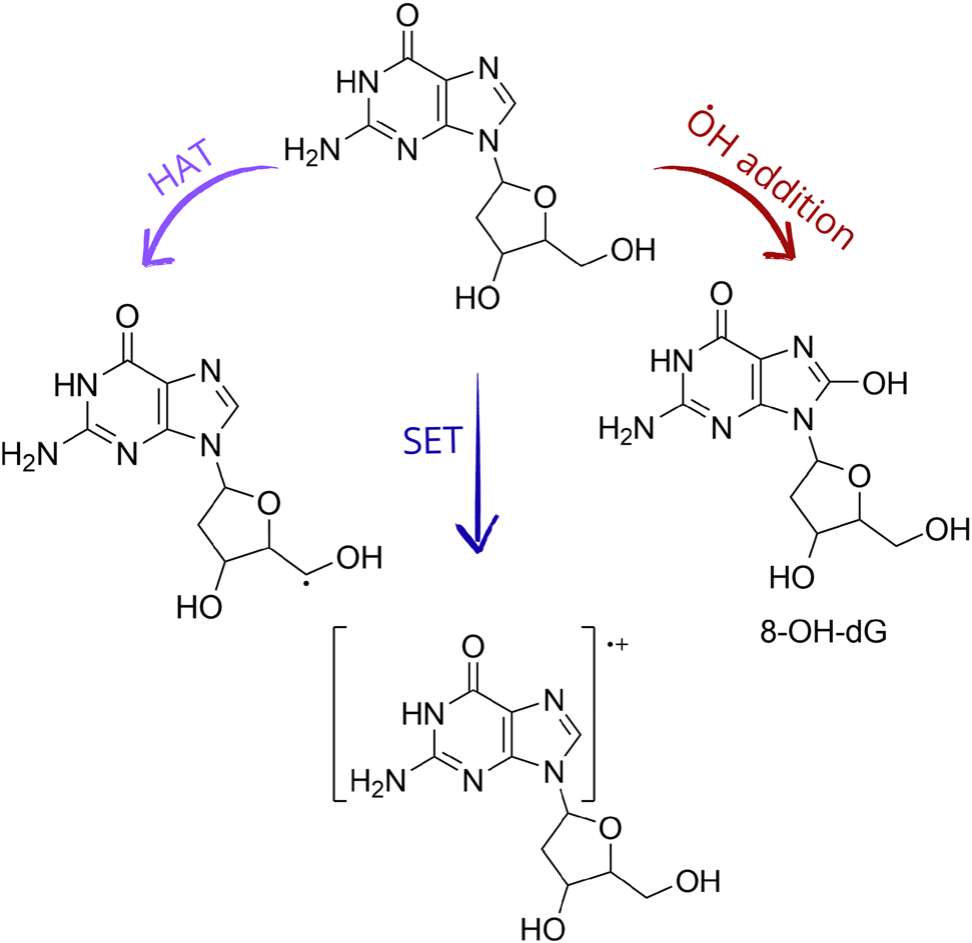
Some of the most relevant oxidation products of 2′-deoxyguanosine (2dG).

In the case of DNA, several reaction mechanisms contribute to its oxidative damage. SET from DNA to oxidants mainly leads to guanine radical cations, since this nucleobase has the lowest redox potential.^82^ This is also the case for 2′-deoxyguanosine, 2dG, and 2′-deoxyguanosine 5′-monophosphate, 2dGMP, within the nucleoside and nucleotide families, respectively.^83^ After oxidative damage, 2dG can rapidly evolve, through deprotonation, to produce C-centered radicals in the sugar moiety,^84^ which are also formed by direct *f*-HAT by ˙OH.^85^ These radicals frequently cause strand breaks.^86,87^ Another key oxidation product of DNA is 8-hydroxy-2′-deoxyguanosine (8-OHdG), which is considered an OS biomarker (Scheme 4).^88^

According to the above-discussed information, to repair lipids, antioxidants must transfer back the lost H atom to them. The same applies to those amino acid residues that are damaged *via f*-HAT and to the C-centered radicals formed in the sugar moieties of DNA. Regarding the guanosine radical cation, it can be fixed by antioxidants through an electron transfer. In other words, for antioxidants to be capable of repairing these damaged biomolecules, they must restore a lost particle. However, this implies the hypothesis that the antioxidants would encounter damaged molecules before the antioxidant reacts with other free radicals and also before the damaged biomolecule further reacts, for example, with O_2_. The radical products formed after the antioxidants scavenge free radicals do not act as H or electron donors, but the opposite. They might become a risk for the molecular integrity of biomolecules.

**Scheme 5:**
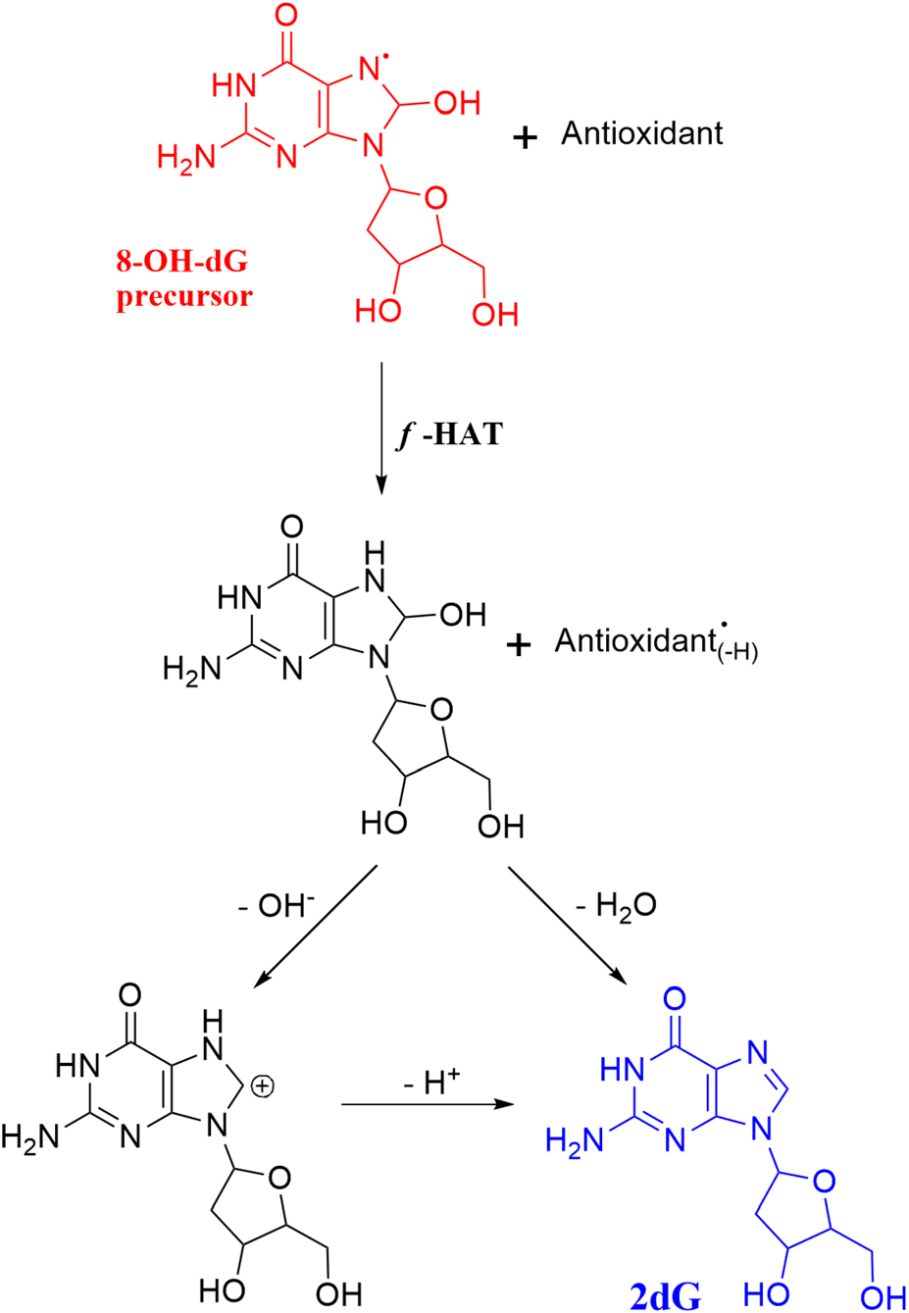
SHATD mechanism, proposed to restore 2′-deoxyguanosine (2dG) from the 8-OH-dG precursor.

Glutathione,^89^ as well as melatonin and some of its metabolites,^90,91^ have been identified as efficient for this purpose when the lost particle is an H atom. Unfortunately, this straightforward strategy does not apply when ˙OH forms adducts in aromatic sites. In the case of DNA, a reaction mechanism named “sequential hydrogen atom transfer dehydration (SHATD)” has been proposed^90^ as a possible chemical route to prevent 8-OH-dG lesions, by repairing its precursor (Scheme 5). On the other hand, a direct antioxidant-mediated repairing route for tyrosine and tryptophan OH adducts, if any, has not been proposed yet.

### 2.4. Promoting enzymatic protection (PEP)

**Fig. 2:**
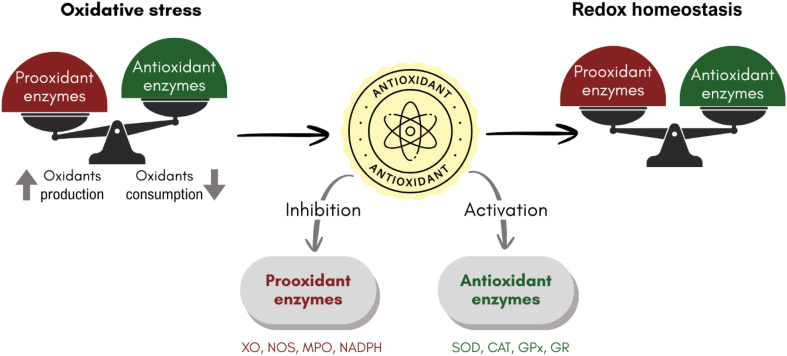
The role of antioxidants in maintaining redox homeostasis.

All the above-mentioned ways of action can be classified as chemical routes. However, antioxidants may modulate the antioxidant enzymatic system, contributing to maintaining the redox homeostasis (Fig. 2). They can inhibit prooxidant enzymes, such as xanthine oxidase (XO), nitric oxide synthases (NOSs), myeloperoxidase (MPO), and nicotinamide adenine dinucleotide phosphate (NADPH) oxidase. Alternatively, they can activate antioxidant enzymes, such as superoxide dismutases (SODs), catalases (CATs), glutathione peroxidases (GPXs), and glutathione reductase (GR). Details on their isoforms, types, and functions can be found elsewhere.^92,93^

There is evidence of antioxidants that modulate the enzymatic system. Melatonin and its metabolites were reported to stimulate antioxidant enzymes and suppress prooxidant enzymes.^94^ Melatonin enhances the expression of SOD1 (copper zinc superoxide dismutase), SOD2 (manganese superoxide dismutase), GPx, CAT, and GR.^95–98^ In addition, melatonin and AMK inhibit the NOS activity.^99^ Curcumin and resveratrol were found to increase the activities of SOD, GR, and glutathione-*S*-transferase (GST) in mice with lung carcinogenesis.^100^

Some nutraceuticals, such as polyphenols, increase the expression of SODs and GPx, while some peptides from food activate CAT.^101^ A pyranoglucoside, from *Euonymus laxiflorus* Champ, referred to as Wal, inhibits the activity of XO, NADPH, CAT, lipoxygenase (LO), and cytochrome P450 (CP450).^102^ Epigallocatechin gallate (EGCG), found in green tea, exhibits a polygenic enzymatic antioxidant action. It downregulates NADPH, XO, COX-2, and LO and upregulates SOD, CAT, GPx, and GST, among other effects.^103^ Gallic acid also inhibits the activity of XO, and theaflavins have similar impacts on MPO, lipoxygenases (LOXs), and cyclooxygenases (COXs).^104^ They also enhance the activity of SOD and CAT.^105^ Isothiocyanates transcriptionally activate antioxidant enzymes such as GST, GPx, and GR.^106^

The role of some medical drugs as modulators of the antioxidant systems has also been investigated. The evaluation of antipsychotics revealed that clozapine increases the activity of SOD1, SOD2, GR and GST, while reducing the CAT activity. Sertindole upregulates both SODs, and ziprasidone decreases CAT activity.^107^ Erectile dysfunction drugs sildenafil, tadalafil, and vardenafil were found to lower the levels of free radicals and OS in rabbits, although they downregulate GST, GPx, and GR. This was attributed to the activation of CAT and SOD activities.^108^ Fluoxetine, an antidepressant, increases the levels of SOD and GST in arthritic rats.^109^

## 3. Computational tools in antioxidant research

**Fig. 3:**
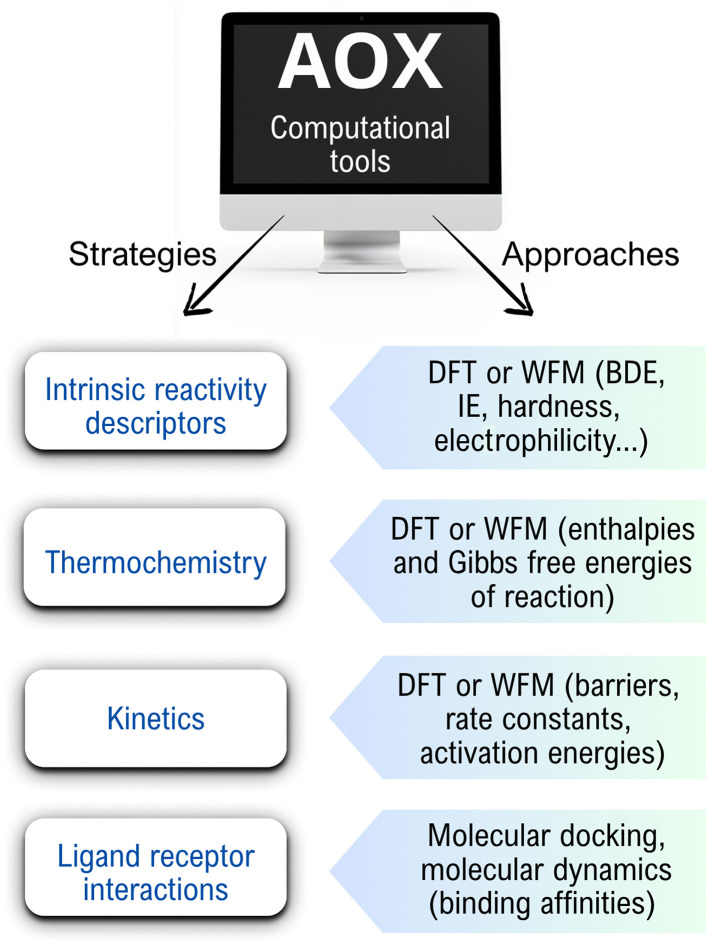
Some of the approaches and strategies most frequently used in computational chemistry to explore AOX. DFT = density functional theory; WFM = wavefunction methods.

The ways of action and reaction mechanisms involved in the antioxidant activity of chemical species can be investigated using different computational tools. Some of the approaches and strategies most frequently used in computational chemistry to explore AOX are shown in Fig. 3. They will be further discussed in this section and contribute to deeper insights into such processes, including chemical structure information, reaction mechanisms, thermochemical feasibility, kinetics, and product distribution information.^110^

Regardless of the used approach, it is essential to use references, or thresholds, that help put the calculated data into perspective. As reliable as computational calculations usually are nowadays, correctly interpreting the numbers obtained from them often requires comparisons to validate predictions. Such comparisons may be performed against experimental data. Moreover, it is always desirable to validate computational approaches or protocols using experimentally measured values. Unfortunately, such data are not always available. However, the errors associated with a particular level of theory (method/basis set) are expected to be rather systematic for similar systems. Thus, if there is no experimental data available for the task at hand, there are (at least) two other options: (A) to validate the calculations using a similar system, for which experimental data are available; (B) using a known reference molecule to compare with and perform calculations for it at the same level of theory. One example of option B, within the antioxidant context, is using Trolox as a reference. Then, the calculated values could be analyzed relative to Trolox. In addition, one of the advantages of using theoretical approaches is their predictive value. Computational tools allow obtaining information that is not known yet or that is very difficult to obtain from experiments. For example, when the investigated systems involve intermediates with very short lives or new designed molecules that have not been synthesized yet.

Theoretical reference data can be used as well. For example, by reevaluating some of the calculations at a high level of theory and supporting the performance of the chosen method *vs.* the results obtained with the refined one. Since the balance between accuracy and time-consumption remains a challenge for computational studies, it might become unfeasible using an accurate level of theory for all the calculations, especially if the investigating reactions occur in complex environments or for molecular systems of relatively large size. Another type of comparison typically used to establish trends in reactivity and add reliability to calculations involves comparing a set of similar molecules that were all calculated using the same methodology. The errors associated with any level of theory are generally alike for analogous systems. Thus, relative values derived from computation are commonly more reliable than absolute ones.

### 3.1. Intrinsic reactivity descriptors

Estimation and interpretation of intrinsic reactivity descriptors is perhaps the most widely used computational strategy to investigate antioxidant activity when it takes place *via* FRS. Bond dissociation energies (BDEs) and ionization energies (IEs) are directly related to the *f*-HAT and SET mechanisms, since they account for H-bond strength and electron donor ability, respectively. Thus, they allow for anticipating trends in H- or electron-donating feasibility among a series of compounds. The AOX for alkyl gallates has been analyzed based on different descriptors, depending on the reaction mechanism: BDE for *f*-HAT, IE and proton dissociation enthalpy (PDE) for SET-PT, and proton affinity (PA) and electron transfer enthalpy (ETE) for SPLET reactions.^111^ The AOX of naringenin and some of its C3-substituted derivatives was also studied based on BDE, IE, and PA.^112^ BDE and IE, together with PDE, were used to predict the antioxidant protection of novel sunscreen-active compounds derived from resveratrol, avobenzone, and octinoxate, *via f*-HAT and SET-PT.^113^ These are only some examples of abundant studies in the literature that take this approach to anticipate AOX.^114–138^ It is important to mention that the accuracy of the methodology chosen for estimating these descriptors is critical. Within the density functional theory (DFT) framework, the LC-ωPBE, M06-2X and M05-2X approximations were recommended to predict BDE, IE and PDE, in different solvents, based on comparisons with the results of CBS-QB3 calculations.^139^ For a series of polyphenols, LC-PBE is recommended for O–H BDEs in the gas phase and LC-PBE, M06-2X, and M05-2X for predictions in aqueous solution, based on the lowest mean absolute deviation *vs.* another accurate methodology: DLPNO-CCSD(T).^140^ For IE, relative low errors have been reported for local pair natural orbital (LPNO) methods and M06-2X,^141^ M11, CAM-B3LYP, ωB97,^142^ and B3LYP.^143^ However, it has been pointed out that the accuracy of the results depends on the molecular size.^143^ Other approaches that are reliable for BDE and IE calculations are the “property-specific atom-centered potentials” (PS-ACPs)^144^ and the electron propagator theory,^145^ respectively.

When analyzing *f*-HAT in terms of BDE, it is implicitly assumed that the Evans–Polanyi principle^146,147^ is fulfilled. It involves a linear relationship between the activation energy and the enthalpy of elementary reactions. Although this is not always the case, and this principle might be theoretically questionable, in some cases, it seems to work nicely. Foti *et al.*^148^ found an excellent correlation between the activation energy (*E*_a_) and the Ar–O BDE (ranging from 77 to *ca.* 86 kcal mol^−1^) of the DPPH radical reactions with 14 phenols. However, the same authors doubt the existence of true linearity for this or any other family of compounds outside small energy ranges. There are other studies supporting the non-linear correspondence between AOX and BDE for the reactions between polyphenols and DPPH.^149^ This was attributed to mechanisms other than *f*-HAT contributing to the overall activity, which also means that BDEs are not enough to describe AOX for these systems. Structural features can also lead to deviations from the Evans–Polanyi principle. A bimodal behavior was found in the rate–BDE correlation for *f*-HAT reactions from the C–H bonds of a large series of substrates to the cumyloxyl radical. Two separated correlations arose from the data analyzed in that study: one for unsaturated hydrocarbons and another for saturated ones.^150^ In addition, Cavus^151^ has pointed out that there are factors that affect experimental results that cannot be fully incorporated into computational models. Attention was called to the possibility that this limitation may lead to erroneous conclusions when AOX is interpreted in terms of BDE, even for the *f*-HAT reaction.

Several reasons might be envisioned to justify why reactivity descriptors fail to predict AOX, and some of them are:

(a) Various reaction channels contribute to the overall reactivity of molecule + free radical reactions, not all of them necessarily corresponding to *f*-HAT.

(b) H-bond-like intramolecular interactions in the transition state, involving the reacting site.

(c) Significant hydrogen atom tunneling effects.

(d) Solvent (particularly water) mediating the H transfer.

(e) Concentration effects influencing reactivity.

(f) The thermochemistry and kinetics of molecule + free radical reactions depend not only on the reactivity of the molecule but also on the reactivity of the reacting radical.

In addition, Bâldea^152^ has recently pointed out that IP, BDE, PDE, ETE, and PA are not independent. The author also formulated two theorems that help interpret the results obtained when these properties are calculated:

• Theorem 1 states that the sums of enthalpies for the SET-PT and SPLET steps are equal: IP + PDE = PA + ETE, since the reactants and products of both mechanisms are identical.

• Theorem 2 states that any part of the identity from Theorem 1 minus BDE = IPH > 0, where IPH is the ionization potential of the H-atom in the medium considered in the calculation.

Bâldea also warned about the reliability of proposing main FRS pathways based only on reactivity descriptors.^152^

The above-discussed issues do not mean that reactivity descriptors cannot be used to investigate AOX. Still, they certainly mean that the results obtained from this strategy should be interpreted with caution. Some efforts have been made to combine more than one descriptor associated with different reaction mechanisms, and to address point (a), keeping fast predictions of potential AOX. This is particularly useful when large sets of molecules are analyzed to identify the best antioxidant candidates. However, more refined studies are strongly recommended to make reliable assessments about the antioxidant activity of any candidate molecule.

**Fig. 4:**
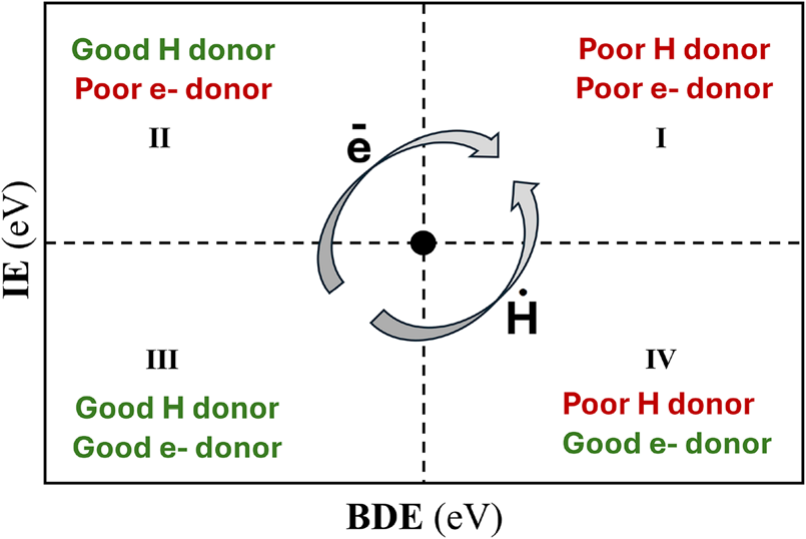
The “electron and hydrogen donating ability map for antioxidants” (eH-DAMA).

One of these attempts is the “electron and hydrogen donating ability map for antioxidants” (eH-DAMA)^153^ (Fig. 4). It is a graphical tool that simultaneously accounts for *f*-HAT and SET, using BDE and IE values, respectively. In this map, the location allows predicting which chemical species is expected to act as an H or electron donor and which one as an acceptor. The map can be used for different purposes, including: (i) analyzing free radical damage to biomolecules, (ii) FRS activity, and (iii) RDB process. Depending on this, the reference species (RS) placed at the black dot in Fig. 4 changes. For purpose (i), RS would be the biomolecule of interest (a lipid, for example), and a variety of oxidants can be incorporated in the map to predict which of them are likely to be damaging *via f*-HAT or SET. For purpose (ii), RS would be the free radical of interest (a peroxyl radical, for example), and diverse antioxidant candidates can be added to map to anticipate which of them would be more effective for scavenging this particular radical, through one or both of these chemical routes. In this case, it is recommended to include some reference molecules that are already recognized as antioxidants (for example, Trolox, ascorbate, or α-tocopherol). For purpose (iii), RS would be the damaged biomolecule, and the relative location of the candidates would allow predicting if they are likely to repair it by restoring the lost electron or the lost H atom. Using FRS, (ii), to illustrate the interpretation of a candidate antioxidant location on the map would be as follows:

• Quadrant I (poor H donor, poor e-donor): it is not expected that the candidate could scavenge the free radical located in the black dot by *f*-HAT or by SET.

• Quadrant II (good H donor, poor e-donor): It is expected that the candidate could scavenge the free radical located in the black dot by *f*-HAT, but not by SET.

• Quadrant III (good H donor, good e-donor): It is expected that the candidate could scavenge the free radical located in the black dot by both *f*-HAT and SET.

• Quadrant IV (poor H donor, good e-donor): It is expected that the candidate could scavenge the free radical located in the black dot by SET, but not by *f*-HAT.

It should be noted, however, that acid/base speciation needs to be considered. For example, it might be possible that a candidate has significant amounts of neutral and anionic species, at the pH of interest. In that case, it is also possible that the neutral form is located in quadrant II and the anion in quadrant IV. Thus, under those pH conditions, the candidate may scavenge free radicals by both chemical routes, depending on the reacting species, *i.e.*, the neutral form *via f*-HAT, and the anion *via* SET.

**Scheme 6:**
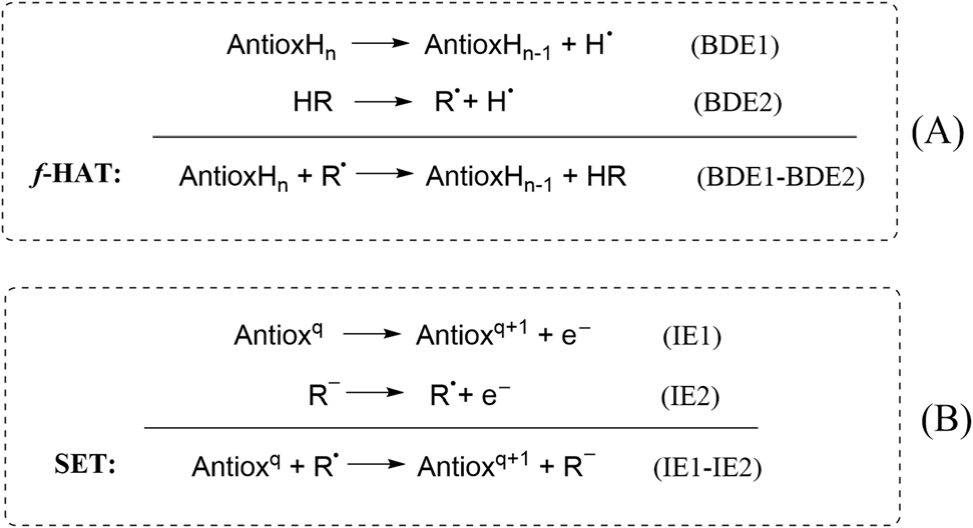
Hess's law expressions for (A) *f*-HAT and (B) SET.

There are relevant aspects of the eH-DAMA tool that deserve some consideration:

The plotted IE and BDE values are not the conventional ones, but those calculated adiabatically and in aqueous solution. Thus, they are directly related to the thermochemical viability of the *f*-HAT and SET reactions, *i.e.*, eH-DAMA is based on the Hess law (Scheme 6).

The calculations are performed in aqueous solution because this is the only biologically relevant medium where SET is likely to occur.

When RS is a free radical (R˙), it is located using the IE of the corresponding anion (R^−^), and the BDE of HR (for example, HOOH, if RS = ˙OOH). This is because there is an implicit competition for the electron between the electron-donating ability of the antioxidant and R^−^ in the SET mechanism (Scheme 6). It would be thermochemically feasible only if IE(antioxidant) <IE(R^−^). A similar reasoning applies to the *f*-HAT mechanism. In this case, the competition involves the H-bond strength of the antioxidant and the HR species, and it would be thermochemically feasible only if BDE(antioxidant) <BDE(HR).

The SPLET mechanism can also be analyzed using the eH-DAMA tool. Since its first step is controlled by the relationship between the *p*Ka of the antioxidant and the pH of the solution, it is not included in the map. The second step is, provided that the deprotonated species of the antioxidant is the one plotted in it.

It is important to keep in mind that molecules that oxidize easily may react with O_2_. If unstable, when exposed to air, they won't be suitable as antioxidants. Thus, comparisons between the electron donor capability of O_2_ and the antioxidant of interest are recommended.

The eH-DAMA tool can be easily modified to include other ways of action and reaction mechanisms involved in the AOX of chemical species.

**Fig. 5:**
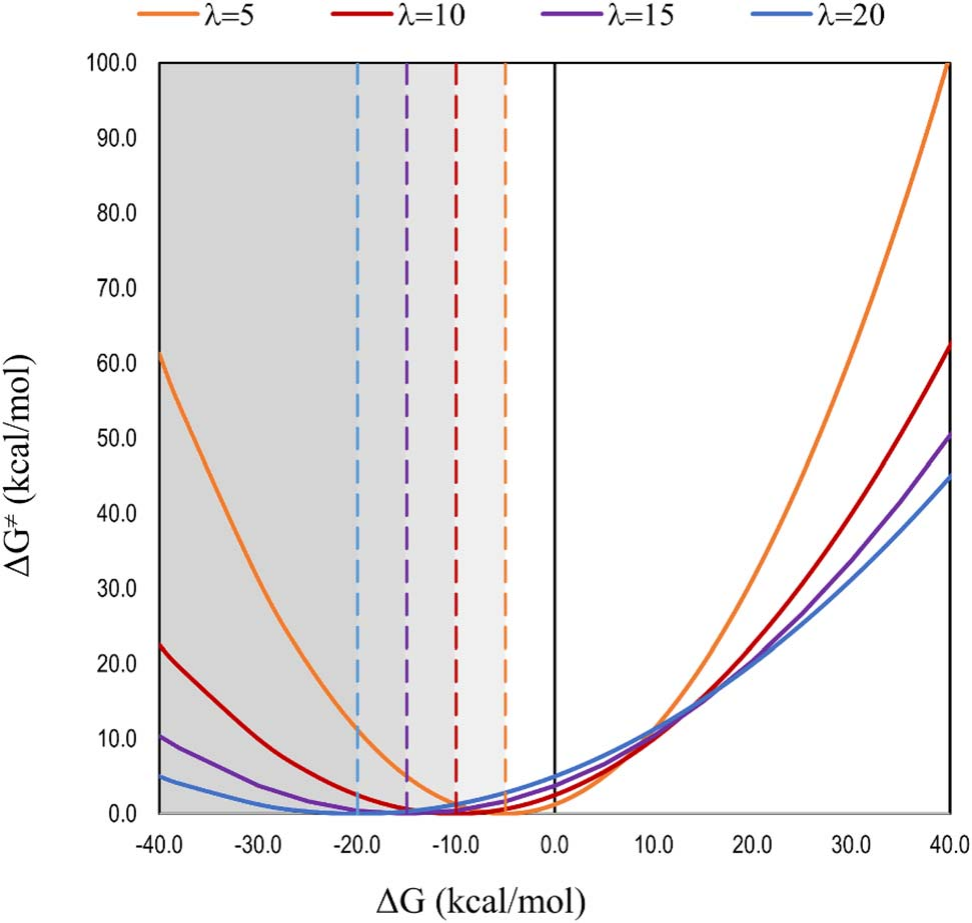
Marcus parabola, plotted for different values of the reorganization energy (*λ*). The values in the legend are in kcal mol^−1^. The dashed vertical lines mark the inversion point of the parabola.

Another consideration deserves to be discussed in more detail: the applicability of IE as a reliable criterion for anticipating SET feasibility. Although counterintuitive, there is not always a direct relationship between thermochemical feasibility and kinetics for this kind of reaction; and if kinetics is too slow, the relevance of a reaction might be negligible, even if largely exergonic. The Marcus parabola (Fig. 5) clearly shows this for electron transfer reactions. It accounts for the relationship between the reaction barrier (Δ*G*^≠^) and the free energy change (Δ*G*) associated with electron transfer reactions: Δ*G*^≠^ = (*λ*+Δ*G*)^2^/4*λ*. Here, *λ* represents the nuclear reorganization energy after the electron is transferred, which involves the inner-sphere reorganization energy (changes in the reactant geometries when they become products) and the outer-sphere reorganization energy (the solvent reorientation around the products to stabilize the new charge distribution). The right part of the parabola follows chemical intuition, *i.e.*, Δ*G*^≠^ increases with Δ*G*. The left part of the parabola, known as the inverted region,^154^ in contrast, implies that Δ*G*^≠^ increases as Δ*G* decreases. The inversion point, *i.e.*, the vertex of the parabola, depends on the reorganization energy and is located at Δ*G* = −*λ*. Thus, the eH-DAMA tool, or any other computational approach, based on IE values as a descriptor for SET reactions, would be reasonably valid only for Δ*G* ≥ −*λ*. Otherwise, the data might be misinterpreted.

**Fig. 6:**
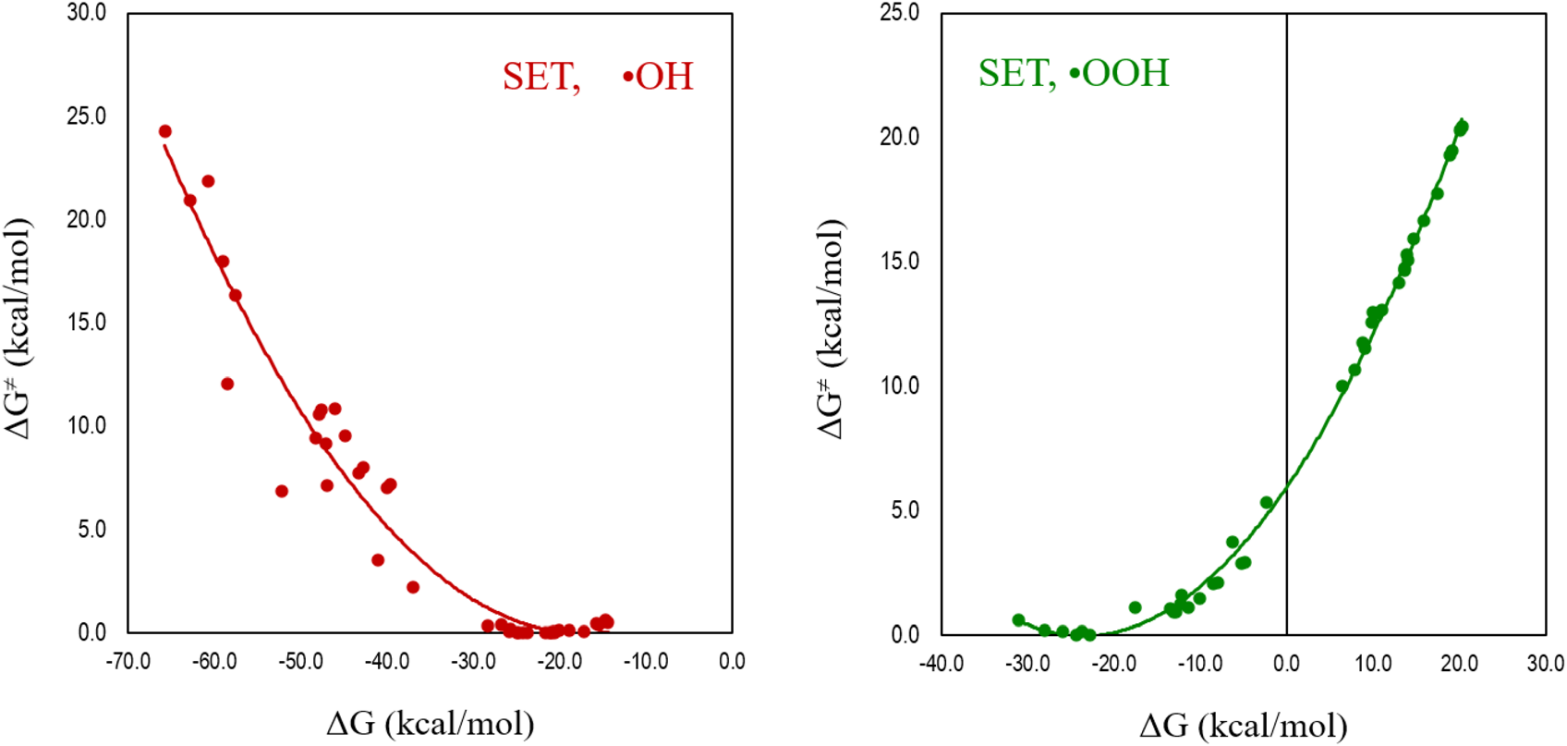
Relationship between the Gibbs energies of activation (Δ*G*^≠^) and the Gibbs energy of reaction (Δ*G*) for the reactions of a series of melatonin derivatives with ˙OH and ˙OOH.

The radical chosen as the antioxidant's counterpart in the studied SET reaction significantly influences the relationship between Δ*G*^≠^ and Δ*G*. The more reactive the radical is, the more likely the reaction is in the inverted region of the Marcus parabola. To illustrate this, the relationship between Δ*G*^≠^ and Δ*G* for the reactions of a series of computationally designed melatonin derivatives^155^ with ˙OH (highly reactive) and ˙OOH (moderately reactive) is plotted in Fig. 6. It clearly shows that while the relationship between Δ*G*^≠^ and Δ*G* is direct for the reaction with ˙OOH, it is inverted for the reaction with ˙OH. There are, at least, two aspects that arise from these results that are relevant to computationally investigating AOX. It is essential to consider the free radical, and not only the intrinsic reactivity of the potential antioxidant. IE cannot always be directly related to the efficiency of a molecule as an electron donor; it would be misleading if the reaction corresponds to the inverted region of the Marcus parabola.

Although the data plotted in Fig. 6 were obtained from calculations, there is experimental evidence on the inverted region of the Marcus parabola. Miller, Calcaterra and Closs were the first to observe this phenomenon, using donor–acceptor organic molecules.^156^ It has also been observed for other organic systems,^157,158^ inorganic systems,^159^ proteins,^160–162^ photo-induced bimolecular electron–transfer reactions in viscous ionic liquid media,^163^ and solid-state molecular junctions.^164^ Probably, the most closely related to the antioxidant context is the recent, combined experimental–theoretical work that evidences the Marcus parabola inverted region in concerted proton-electron transfer reactions.^165^

Reactivity descriptors, other than IE, can also be used to predict electron flux in AOX-related research. Some of them are derived from conceptual DFT, such as Mulliken electronegativity, chemical hardness, the electrophilicity index, and the electron-donor and electron-acceptor capacities.^166–173^ The first oxidation potential can also be used for that purpose.^174^ There are also descriptors, other than BDE, that can be related to H-bond strength. The *δg* descriptor from the “independent gradient model” quantifies the electron density gradient and can be used to analyze both covalent and non-covalent interactions.^175^ The “intrinsic bond strength index” allows for the ranking of two-center bonds in a molecular environment by their intrinsic strength.^176^ The “interaction region indicator” facilitates the comparison of the relative importance of *f*-HAT and SET mechanisms on the AOX of chemical compounds.^151^ All of them can contribute to gaining a more complete picture of the complex chemistry of antioxidants.

### 3.2. Thermochemistry of reactions in AOX

Identifying if chemical reactions involved in AOX are expected to occur spontaneously under relevant physiological conditions is crucial to predicting the antioxidant behavior of chemical compounds. Moreover, calculating thermochemical data (such as Δ*H* and Δ*G*), as the energy difference between products and reactants, for the reactions involved in AOX solves some of the limitations of reactivity descriptors. This strategy allows for the concurrent investigation of several mechanisms and reaction channels. Solvent effects can be included, as well as acid–base speciation at the pH of interest, provided that the p*K*_a_ values of the studied species are known. The radical counterpart of the antioxidant is explicitly considered, and temperature effects are included in the calculations *via* statistical thermodynamics. All the ways of action discussed in Section 2 of this review can be investigated using this approach.

Thermochemical analysis of AOX reactions has been used to predict the relative importance of *f*-HAT, SPLET, and SPET mechanisms in the HOO˙ scavenging activity of a series of chalcone derivatives^177^ (FRS way of action); to assess the feasibility of repairing α-, γ-, and δ-tocopherol radicals by *f*-HAT from l-ascorbyl palmitate^178^ (RBD way of action); to evaluate ferulic acid as Fe(ii) and Fe(iii) chelators, as well as the possible prooxidant effect of the formed Fe(iii) complex when reduced by ascorbate^179^ (IOP way of action). It has also been used to predict if C-centered radicals, of a set of hydrocarbons, are likely to react with O_2_, a pathway involved in ROO˙ formation.^180^

However, as rightfully pointed out by Mulder *et al.*,^181^ two decades ago, the prediction of promising antioxidants from quantum-thermochemical calculations must be done with caution. This is because several aspects of their chemistry should be taken into account before drawing conclusions from such predictions. These authors specified properties that molecules must have to be effective and safe antioxidants. Some of them are:

(1) The rate constant for their *f*-HAT reaction with peroxyl radicals (ROO˙) must be significantly higher than the reaction of ROO˙ with lipids.

(2) The products yielded by the molecule + ROO˙ reactions should not react with O_2_, otherwise a new peroxyl radical is formed and may propagate the oxidation.

(3) The products yielded by the molecule + ROO˙ reactions should not react with lipids either, for the same reason.

(4) The investigated molecule, and the products formed after its reaction with free radicals, should not be toxic.

Point 1 highlights the importance of considering kinetics for reliable predictions of AOX. The following section is devoted to detail this aspect. Points 2 and 3 are related to the possible prooxidant effects of molecules intended as antioxidants. An example of a possible way to compare these two opposite activities for phenols can be found in ref. 182. While this is a crucial aspect to consider when proposing a chemical compound as antioxidants, points 2 and 3 have been scarcely explored using computational tools. Reactions of biomolecules with the radicals formed after the FRS processes should also be considered as a potential source of peroxyl radicals *in vivo* (point 2). Regarding toxicity (point 4), some descriptors, including IC_50_ and Ames mutagenicity, can be estimated using software based on “Quantitative Structure–Activity Relationship” (QSAR). Other routes would require quantum mechanical approaches, including the one mentioned in point 3, which can be considered as a toxic effect. If radicals produced by antioxidant candidate + free radical reactions are still capable of damaging lipids, or any other biomolecule, such a candidate cannot be considered an efficient antioxidant. Although this undesirable effect has been considered for phenolic compounds,^182^ there is still a lack of computational studies on it. There are also other risks that phenols might pose to the chemical integrity of biomolecules, including protein arylation,^183^ another aspect of their chemistry frequently overlooked. The likelihood of all of them can be predicted with the same computational tools used to predict AOX. To that purpose, it is recommended to use kinetics-based approaches since, in biological environments, competing reactions would determine the fate of chemicals and their potential benefits or risks.

There is still a point, other than those specified by Mulder *et al.*, that deserves to be mentioned.

(5) The investigated molecule should not react with O_2_. If it does, it will be unstable under air conditions. In addition, it might also react with O_2_ under physiological conditions before reaching the intended target.

Another aspect worth mentioning is that, when thermochemistry is used to predict the relative feasibility of different reaction mechanisms, it is recommended to do so using Δ*G*: The quantity includes entropic effects that may be crucial when comparing the RAF mechanism with any of the others shown in Scheme 1. The entropic loss associated with the formation of the addition product may lead to an inversion of the conclusions drawn based on Δ*G* compared to those using Δ*H*, particularly when the comparison involves, for example, RAF and *f*-HAT.

Solvent effects should also be included in the calculations. The most widely used approach, due to its computational efficiency, especially when combined with DFT approaches, is to use an implicit solvent model. Some examples are: the “universal solvation model based on solute electron density” (SMD),^184^ the “polarizable continuum model” (PCM),^185,186^ and its various formulations including the “integral-equation-formalism” (IEF-PCM),^187–189^ Two different media are relevant to AOX: lipid (aprotic, non-polar) and aqueous (protic, polar). The polarity of the solvent determines the attainable concentration of the potential antioxidant, based on its solubility, and modulates its AOX. Although not always observed, a phenomenon known as the “polar paradox” may arise. It has been described that polar molecules behave as better antioxidants in oily systems, while the AOX performance of non-polar ones is better in water/lipid emulsions.^190^

In aqueous solutions, on the other hand, pH influences the reactivity of molecules that exist in acid/base equilibria. A very well-known case is the SPLET mechanism, proposed by Litwinienko and Ingold.^47,191^ The pH would influence any reaction that involves protonation/deprotonation processes, for example, the CDCM mechanism described in Section 2.2:M(ox) + H_2_L → M(ox)–LH + H^+^

If the pH is not considered in the calculation of the corresponding Δ*G*, it corresponds to standard conditions (Δ*G*^0^), *i.e.*, a 1 M concentration for all species, which means pH = 0. This is not a pH found in biological media. Thus, it is not relevant in the AOX context. However, for any particular pH of interest, a conditional Δ*G* (Δ*G*′) can be obtained from the corresponding conditional equilibrium constant.^192,193^ The relationship between them for the above-described CDCM reaction would be: Δ*G*′ = Δ*G*^0^ − 2.303RT(pH).^69^ This implies that, in this example, increasing the pH would promote the exergonicity of the reaction (at 298.15 K and pH 7.4, Δ*G*′ becomes 10.1 kcal mol^−1^ lower than the value under standard conditions). A similar approach can be used for any other reaction involving proton exchange. In this way, the pH effect is included in the thermochemical calculations.

### 3.3. Kinetics and reaction barriers *vs.* rate constants

Let's start this section by differentiating between barriers (Δ*G*^≠^) and activation energies (*E*_a_). Barriers are the energy differences between the transition state (TS) and the reactants of any elementary reaction that contributes to the transformation of reactants into products. Activation energies, on the other hand, are unique for the overall reaction, at any given temperature (*T*), even when it involves several elementary steps. While barriers always range between zero and a positive energy value, activation energies can be positive (when the rate increases with *T*), or negative (when the rate decreases with *T*). Although the latter case might seem counterintuitive, it is relatively common in molecule–radical reactions.^194–204^ This is frequently due to non-elementary, two-step, reactions that involve the formation of a reactant complex.

Evaluating kinetics from theoretical approaches involves locating the TS, calculating the corresponding Δ*G*^≠^, and obtaining *k* for elementary reactions. Suppose the reaction takes place through a multi-step mechanism. In that case, this procedure is followed for each relevant pathway, and the individual *k* values are combined to estimate the overall rate constant (*k*^overall^). There are several theories that allow calculating *k* for bimolecular reactions, which is the case for most of the chemical processes involved in AOX, for example, conventional Transition State Theory (TST),^146–206^ and Variational Transition State Theory (VTST) in its various implementations.^207–209^ However, to properly do so, it is crucial to know, or to propose, the mechanism associated with the reaction of interest. It would determine the proper way to combine the *k* value of each step into a *k*^overall^ single constant. In addition, while TST is usually reliable enough for investigating reactions with significant barrier heights, VTST is a better option for barrierless reactions. VTST is always more accurate but also more computationally demanding. Thus, from a practical point of view, at least for systems as complex as those involved in AOX, it may be a reasonable choice to use VTST only when it is necessary. If the same method, basis set, and solvent model are used, it is possible to obtain the rate constant for some reaction pathways with TST and others with VTST, and then combine them to estimate the *k*^overall^.

In contrast, experimental techniques typically involve measuring concentration changes over time (*t*). The reaction rate (*ν*) is obtained from the concentration *vs. t* plot. To that purpose, initial reactants or final products can be used (*ν* = −d[React]/d*t*) or *ν* = d[Product]/d*t*, respectively). To obtain the rate law (for example,*ν* = *k*[Reactant_1_]^*m*^[Reactant_2_]^*n*^), the experiments are repeated, varying the initial concentrations and estimating the corresponding initial rates, *ν* (*t* = 0). Then, *k* is calculated from the rate law expression using the concentration values. Since *E*_a_ reflects the influence of temperature (*T*) on the reaction rate, trials must be repeated at various temperatures to obtain *k* = *f*(*T*). Then, *E*_a_ is extracted from *k* = *f*(*T*). Different equations can be used for that purpose, including the Arrhenius equation.

Accordingly, the magnitude directly comparable with experimental values is *k*^overall^. Thus, since antioxidant-related mechanisms often involve multiple, competing pathways, the most likely situation is that the theoretically calculated Δ*G*^≠^ value for any individual pathway won't be directly comparable with activation energies derived from experiments.

This point has only been briefly addressed because the determination of rate constants from experimental data falls outside the scope of this review. Information about this topic can be found in kinetics textbooks. The aspects mentioned above were included here to illustrate why the calculated Δ*G*^≠^ and *E*_a_ are not necessarily comparable. They might have the same value if the studied reaction is elementary and has no significant quantum effects, like tunneling. Only in such cases, the calculated Δ*G*^≠^ is expected to agree with *E*_a_ values from experiments. Therefore, for bimolecular reactions involving more than one relevant pathway, which is the case for most AOX mechanisms, calculating the *k*^overall^ is probably the best approach to make reasonable predictions about the potential antioxidant performance of the investigated candidates. Since the most challenging aspect of investigating kinetics using computational tools is arguably locating the transition states, this author sees no reason why not to proceed to estimating the rate constants once the Δ*G*^≠^ values are obtained.

Some key aspects to consider when pursuing that goal have been detailed for the computational protocol known as QM-ORSA (“Quantum Mechanics-based test for Overall free Radical Scavenging Activity).^210^ They are summarized next.

All the possible mechanisms involved in the AOX of a candidate antioxidant should be considered. The number would depend on the structure of the candidate and on its acid–base equilibria in aqueous solution, at relevant pHs.

The thermochemistry calculated using the available computational codes for electronic structure calculations, including Gaussian, is reported in 1 atm standard state. Since most of the reactions in AOX pathways are bimolecular, and this activity occurs in solution, the Δ*G*^≠^ values should be converted to the 1 M standard state.

For pathways involving hydrogen transfer, tunneling effects should be included in the *k* calculation. Different approaches can be used to do this, including the Eckart,^211,212^ the zero-curvature tunneling^213^ (ZCT), and the small-curvature tunneling (SCT)^214^ methods.

The reaction path degeneracy should be considered, if present. It can be obtained using, for example, the strategy proposed by Pollak and Pechukas.^215^

The calculated *k* values should be corrected when they are within the diffusion-limited regime. Otherwise, they would lack physical meaning, *i.e.*, *k* values larger than the diffusion rate would mean that the reaction occurs faster than the reactants encounter.

The rate constant of each exergonic pathway should be included in the *k*^overall^ calculation, weighted by the molar fraction of the corresponding acid–base species of the candidate, at the pH of interest, if the reaction is studied in aqueous solution.

Establishing a threshold value helps identify candidates with significant AOX. When it takes place through FRS, the *k* of the ˙OOH + PUFA reaction can be used for that purpose. This would be in line with point 1 proposed by Mulder *et al.*^181^ However, in that case, the radical used in our modeling should be the same.

The experimental rate constants for the ˙OOH + PUFAs reaction can also be used to validate the computational methodology used. They have been measured to be 1.18 × 10^3^ M^−1^s^−1^ and 3.05 × 10^3^ M^−1^ s^−1^, for linoleic and arachidonic acids, respectively.^216^ It is worthwhile mentioning that these values were obtained under strong acidic conditions, and under such conditions the hydroperoxyl radical is the main species of the ˙OOH/O_2_˙^−^ acid/base pair, *i.e.*, its molar fraction ∼1. In contrast, at pH = 7.4, for example, such fraction becomes 2.5 × 10^−3^ (*p*Ka˙_OOH_ = 4.8 (ref. 217)), which means that the rate constant for the ˙OOH + PUFAs reaction would be 400 times lower (∼2.9–7.6 M^−1^ s^−1^). Thus, to use these values as references, the ˙OOH fraction, at the pH of interest, should be considered.

To make fair comparisons with reference compounds, like Trolox, it is crucial to use not only the same radical but also the same level of theory (method, basis set, and solvent model). It should also be noted that when DFT is used, which is the most common case, not all its approximations are equally reliable for estimating Δ*G*^≠^. In a benchmark study, the following have been recommended for the kinetics of molecule + free radical reactions in solution, when the SMD model is used to mimic the solvent: LC-ωPBE, M06-2X, BMK, B2PLYP, M05-2X, and MN12SX. They were chosen by comparison with experimental data.

**Fig. 7:**
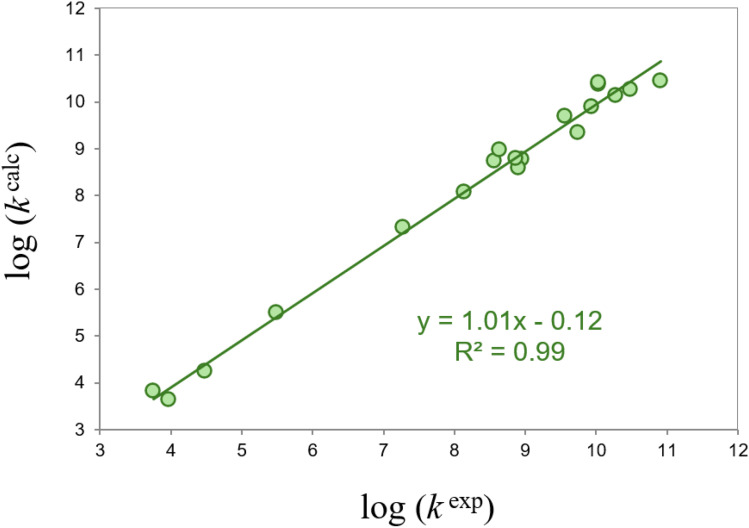
Linear correlation between rate constants obtained using the QM-ORSA, log(*k*^calc^), protocol *vs.* experimental values, log(*k*^exp^). The values correspond to those reported in Table 2.

The QM-ORSA protocol is based on TST, or VTST, theories. It was validated against experimental data^218^ and has been successfully used by its developers^219–224^ and by independent authors^225–241^ to predict AOX through different ways of action. Table 2 shows a comparison between rate constants calculated with this protocol *vs.* experimental data for radical + molecule reactions. The largest discrepancy corresponds to the Trolox + ˙OH, with the calculated value being only 2.9 times lower than the experimental one. The corresponding linear correlation is presented in Fig. 7. As this figure shows, not only *R*^2^ is almost 1 (0.99), but also the slope is (1.01), and the intercept is close to 0 (−0.12). Regarding the IOP mechanism, for the “free” Cu(ii) + O_2_˙^−^reaction, the rate constant calculated with the same protocol (4.7 × 10^9^ M^−1^ s^−1^)^242^ is only 1.7 times lower than the experimentally measured one (8.1 × 10^9^ M^−1^ s^−1^).^243^ These agreements between calculated and experimental data support the reliability of the QM-ORSA protocol.

**Table 2:** Rate constants (M^−1^ s^−1^) calculated with the QM-ORSA protocol, compared to experimental values

Reaction	Calculated	Ref.	Experimental	Ref.
Ascorbic acid/ascorbate + HOO˙/O_2_˙^−^	3.07 × 10^5^	210	3.10 × 10^5^	244
Caffeine + ˙OH	2.15 × 10^9^	245	5.60 × 10^9^	a
Capsaicin + ROO˙	6.50 × 10^3^	246	5.60 × 10^3^	247
Edaravone + ˙OH	1.35 × 10^−10^	248	1.93 × 10^10^	b
Ellagic acid + ˙N_3_	4.95 × 10^9^	71	3.70 × 10^9^	249
Ellagic acid + ˙OOCCl_3_	1.15 × 10^8^	71	1.40 × 10^8^	249
Eugenol + ˙OOCCl_3_	6.16 × 10^8^	250	7.50 × 10^8^	251
Gallic acid + ˙OH	2.56 × 10^−10^	219	1.10 × 10^−10^	252 and 253
Glutathione + ˙OCH_3_	5.89 × 10^8^	254	9.00 × 10^8^	255
Glutathione + ˙OH	7.68 × 10^9^	254	8.72 × 10^9^	c
Melatonin + ˙OH	1.85 × 10^−10^	256 and 257	3.04 × 10^−10^	d
Melatonin + ˙OOCCl_3_	9.20 × 10^8^	256 and 257	4.35 × 10^8^	e
Sesamol + ˙OH	2.37 × 10^−10^	258	1.10 × 10^−10^	259
Sesamol + ˙OOCCl_3_	5.41 × 10^8^	258	8.10 × 10^−10^	259
Trolox + ArO˙	1.72 × 10^4^	50	1.93 × 10^10^	260
Trolox + ˙OH	2.78 × 10^10^	50	1.40 × 10^8^	261
Tyrosol + ROO˙	4.24 × 10^3^	262	3.7 × 10^9^	263
Uric acid + tryptophanyl radical	2.07 × 10^7^		1.90 × 10^7^	
Vanillin + ˙OOCCl_3_	3.83 × 10^8^	250	1.10 × 10^−10^	264

a Average value from ref. 265–267.

b Average value from ref. 268 and 269.

c Average value from ref. 270–272.

d Average value from ref. 273–277.

e Average value from ref. 278 and 279.

It is worthwhile to mention that, when using this protocol, solvent effects are mimicked using the SMD continuous solvent model. Thus, although properly including solvent effects in computational modeling remains a current challenge, the results shown in Table 2 and Fig. 7 suggest that the SMD model properly accounts for them. It uses electron density instead of partial atomic charge and is considered universal because it can be used for any solute (charged or not) in any solvent for which the necessary descriptors are known.^184^ The mean unsigned errors of this model were reported to be of 0.6–1.0 and 4 kcal mol^−1^ for solvation free energies of neutral compounds and ions, respectively. The effects of including explicit solvent molecules when investigating the chemistry of antioxidants still deserve further research. This would be of particular importance for aqueous environments and molecules, and free radicals, that can be involved in H bond interactions.

Regarding the hydroperoxyl radical, its potential role as a reducing agent, and its synergy with antioxidants during lipid peroxidation has been recently reviewed.^280^ The double-faced oxidant/antioxidant role of this radical distinguishes it from other peroxyl radicals. As is previously mentioned, ˙OOH and the superoxide radical anion are interconnected *via* acid/base equilibrium, which favors the latter when pH ≥4.8. It has been hypothesized that, because O_2_˙^−^ is a strong reductant, it can act as an electron donor, repairing oxidatively damaged biomolecules.^281,282^ It has also been proposed that O_2_˙^−^ can regenerate antioxidants with the catechol moiety, *via* SET, allowing them to scavenge several radical equivalents.^48,71,283^

### 3.4. Ligand–receptor interactions

The investigation of the interactions between antioxidant candidates and the receptors involved in the enzymatic AOX defense systems (Section 2.4) is a relatively new and increasingly active area of research.^284^ The computational strategy most used for this purpose is molecular docking, which can be combined with molecular dynamics (MD) simulations. Although these tools have been widely used for other purposes, especially for testing the potential use of newly designed compounds in the treatment of diverse diseases,^285–289^ they are not so commonly used to assess enzymatic AOX.

For protein–ligand interactions (PLI), docking enables the prediction of the most likely binding pose and the corresponding binding affinities (Δ*G*_bind_), which are semi-empirical scores comparable to experimental values when the protocol is refined. This is usually achieved at low computational costs, *i.e.*, in a short time and using modest computational resources. The pose of the ligand in the receptor site helps identify which amino acid residues are involved in the PLI and to characterize each interaction type (H-bonding, hydrophobic, electrostatic, metal-donor, *etc.*). Δ*G*_bind_ is used to quantify the strength of the interaction and to propose promising candidates: The more negative the Δ*G*_bind_ is, the better the candidate. Defining the binding site region is a key requirement for docking calculations. It is usually obtained from an experimental structure, reported in a database, such as the RCSB Protein Data Bank. The same region found there for the interaction between the protein of interest and other ligands is used for the antioxidant PLI. Alternatively, blind-docking strategies or specialized software that search for binding sites can be used.^290–292^ However, defining the optimal interaction region for each specific case remains a challenge.^293^

One recognized weakness of molecular docking is the rigidity of the receptor during the calculations. Thus, conformational changes that arise as a consequence of the PLI are not taken into account, which may lead to inaccurate predictions.^294^ One way to overcome this limitation is to use ensemble-based docking, with experimental^295^ or MD-generated^296^ structures. The “Induced Fit Docking” approach^297^ can also be used for this purpose. Another docking issue is that there is no scoring function (SF) optimal for every system. This can be addressed by using a consensus method^298^ in which multiple SFs are used. The consensus method usually outperforms single-function approaches,^299^ although there are studies showing the opposite trend.^300,301^ The performance of the consensus method is influenced by several factors, including the selection of the combined SFs,^302^ which might lead to artificial enrichment and the consequent high success rates,^300,303^ the ranking strategy,^304^ the number of tested datasets,^305^ and the score parametrization.^306^ More details on docking methodologies are discussed elsewhere.^307–309^

MD can be used independently to assess PLI or to refine docking results. One drawback of MD studies is that they are highly computationally demanding. Thus, the advantage of MD as a post-docking approach is that it can only be used to refute or confirm promising candidates. If confirmed, MD can also provide deeper insights into its interaction with the protein and enhance the predictions' reliability. In addition to incorporating structural flexibility, MD-based calculations enable filtering unstable binding modes;^310^ improving docking poses, binding affinities, and kinetics;^311^ and including the effects of water^312^ and ions^313^ in the PLI. The interested reader is referred to a recent, thorough review on the use of molecular docking and MD to investigate PLI.^314^

Despite their limitations, docking calculations have been successfully used to investigate the PLI in some AOX-related systems. Based on Δ*G*_bind_ values, it has been proposed that vanillin, and to a lesser extent vanillic acid, may offer protective effects against oxidative brain damage.^315^ The calculated CAT affinities of these compounds were supported by *in vivo* experimental evidence. Eugenol, cyclohexane and caryophyllene were predicted to fix oxidative/antioxidative unbalances by downregulating MPO and upregulating SOD, CAT and GPx.^316^ The corresponding Δ*G*_bind_ values were described as comparable to those of prednisolone, in agreement with the results from *in vivo* experiments. Mangiferine was found to bind strongly to CAT, which was confirmed by fluorescence spectroscopy.^317^ The binding of benzothiazole analogs to the CAT catalytic site was estimated to be as strong as that of valproic acid,^318^ a substance used to treat epilepsy. The computational results were supported by experiments showing that these compounds protect neuronal cells from OS-mediated damage, increasing their viability and enhancing CAT's activity during hydrogen peroxide (H_2_O_2_) exposures. The enzyme-mediated AOX of a thiazine–pyridine hybrid was modeled at SOD and CAT active sites. Based on the magnitude of the Δ*G*_bind_, it was hypothesized that this compound may decrease OS by increasing SOD activity.^319^ In addition to its FRS activity, the effects of tocotrienol in alleviating OS conditions were proposed to involve PLI with the antioxidant enzymes GR, CAT, SOD, GST, and GPx. These findings aligned with *in vivo* experiments.^320^ Quercetin's binding to CAT was described as strong, with higher affinity than ascorbic acid towards this enzyme.^321^ The theoretical prediction was confirmed by the experimental observation that CAT activity increased in diabetic rats after quercetin consumption.

On the other hand, PLI involving antioxidant/oxidant enzymes can have the opposite effect. Dexamethasone was described to bind DNA, at the catalytic site in GPx-4, the FAD site in GR, the active site in SOD, and the NADPH residues in CAT.^322^ Based on these results, it was hypothesized that dexamethasone may damage DNA indirectly by inhibiting the antioxidant defense system and increasing OS. This hypothesis aligns with the adverse effects of this glucocorticoid. The structural characterization of the PLI between resveratrol and CAT provided new insights into the AOX of this compound and was corroborated by a variety of experimental techniques.^323^

Regarding combined MD-docking strategies, two examples are discussed next. A series of oxycoumarin derivatives were investigated as potential antioxidants. In addition to their FRS activity, their effects on the antioxidant enzymatic system were also evaluated considering SOD, GST, and CAT.^324^ A docking study was conducted to characterize the binding mode of the compounds in the active site. Different non-bonded interactions were identified, including H-bonding, salt bridge, electrostatic, van der Waals, and hydrophobic interactions. Post-docking MD simulations were performed for the most promising candidate, including “molecular mechanics Poisson–Boltzmann surface area” and “generalized Born surface area” to obtain the solvent contributions to Δ*G*_bind_. The theoretical predictions paralleled the *in vivo* results for lipid peroxidation in rats treated with H_2_O_2_.

The PLI of nine oligopeptides from bovine hemoglobin with the Keap1 (Kelch-like ECH-associated protein (1) receptor was investigated.^325^ The Keap1–Nrf2 (Nuclear factor erythroid 2-related factor (2) pathway plays a crucial role in controlling OS.^326^ Disruption of the Keap1–Nrf2 interaction by exogenous antioxidants regulates the expression of antioxidant enzymes, including CAT, SOD, and GPX.^327^ According to docking results, the investigated oligopeptides bind to the Keap1 pocket, mainly through H-bonding interactions.^325^ For the systems with the strongest PLI, MD calculations confirmed the docking predictions. It was proposed that these peptides are likely to bind to Keap1, preventing NrF2 interactions. Thus, they are expected to activate this transcription factor, promoting the subsequent increase in the expression of antioxidant genes.

Regarding the comparisons recommended at the beginning of Section 3, using natural substrates or reference ligands known to act as agonists/antagonists of the receptors modulating AOX might contribute to increasing the reliability of predictions made with docking and MD approaches. For example, xanthine and oxypurinol can be used as references to evaluate the likeliness of a candidate to act as an XO inhibitor. Xanthine is one of the natural XO substrates,^328^ and oxypurinol is a known XO inhibitor.^329^ If the Δ*G*_bind_ value of an antioxidant candidate is similar to or more negative than that of xanthine, it could indicate a potential for inhibition. If it also surpasses the Δ*G*_bind_ of oxypurinol, the chances of that are even better. However, affinities alone are not enough. The receptor residues and the type of interactions involved in the PLI should also be similar, at least to some extent. If the PLI of the substrate involves the active site, while the inhibitor binds to an allosteric site, this needs to be considered as well. Logically, for those comparisons to be fair, the PLI between XO and all the ligands must be simulated with the same computational approach, and the binding pocket should also be the same.

## 4. Challenges and future directions

The main challenge of using computational tools to predict AOX and establishing trends on this activity arises from the chemical complexity of the processes involving these substances in biological environments. Such complexity is evident in the variety of ways of action that they might exhibit, and the diverse mechanisms involved in each of them, as discussed in the previous sections of this review. Investigating only one of them may not be enough to decide whether a candidate is likely to be an efficient antioxidant or not. Knowledge is frequently gained through small steps. Within the AOX context, this means that several studies may be required to make reliable predictions. Conclusions should be cautiously drawn from analyzing a limited aspect of antioxidants' chemistry. Conclusions should also be specifically stated. A candidate with a low BDE, or good for scavenging free radicals, is not necessarily a good antioxidant; it, or products yielded by its scavenging process, might have prooxidant effects. Any of them can be toxic or metabolized to toxic species, promote ˙OH production through reduction of redox metals involved in Fenton-like reactions, or modulate AOX-related enzymes in an adverse way. The opposite situation is also possible. A candidate with modest FRS may have OS-protective effects through a different route, or its metabolites can be suitable for scavenging free radicals. Another aspect to consider is the variety of oxidants that coexist in biological environments. Attention has been called to the need of including other than oxygenated ones in the modeling.^330^

Computational studies devoted to investigating reactions with O_2_ are still scarce. At least two aspects of such reactions are directly related to antioxidant efficacy: (I) the chemical stability of the antioxidant candidate under air conditions and (II) the possible reaction between O_2_ and the radicals formed after the FRS processes. The second one might produce ROO˙ species still capable of damaging biomolecules, especially lipids. Another phenomenon, rather unexplored so far using computational tools, is the possible synergy between antioxidants. This would be particularly important considering endogenously produced ones, such as glutathione, since they might be present in the same environment as the investigated molecule. Interactions between antioxidants and medical drugs is another area of research that requires further studies. Many OS-related diseases are multifactorial, which means that cocktail-like medications may involve antioxidants and other chemicals. Anticipating the effects of such interactions may help promote safer, more efficient, therapeutic regimes.

Solvation effects and protonation/deprotonation equilibria also modulate AOX. Thus, as challenging as it might be, it is recommended to consider them. The approach most frequently used to mimic solvent effects in electronic calculations is to use continuum solvent models. Although they have been proven to account for solvation rather accurately for a large variety of solutes and solvents, the effects of including explicit solvent molecules when investigating the chemistry of antioxidants still deserve to be explored further. This would be particularly important for molecules and free radicals that are likely to be involved in H bond interactions, in aqueous environments. When using quantum chemical-based approaches, it is unfeasible to include a large enough number of solvent molecules in the calculations, due to time consumption. However, it would be interesting to evaluate the influence of a few explicit water molecules, combined with a continuum model, on the results, for example, comparing calculated *vs.* experimental rate constants. Finding a way to include concentration effects in computational approaches is still an unsolved challenge, related to this topic.

Additionally, as with any other technique, uncertainties are associated with any level of theory used in computational chemistry. Therefore, validating the approach used is important to support the reliability of the predictions. The major challenge of theoretically predicting AOX can be summarized as follows: they require a picture as complete as possible of the overall chemistry of a candidate to propose it, within a reasonable level of certainty, as an antioxidant viable to reduce OS in living systems.

It has been previously proven that single-property-based analyses are far from enough to describe the AOX of flavonoids.^331^ Not even their FRS activity can be accurately predicted using individual properties. A map that simultaneously displays various descriptors was proposed to visualize the antioxidant behavior of these compounds more comprehensively. An all-encompassing view of the complex landscape associated with the AOX of quercetin is also provided in that work. It clearly shows the interconnection and diversity of properties influencing such activity, including structure, thermochemistry, kinetics, PLI, and bioavailability. Future efforts in this direction are expected to enhance our understanding of AOX and to make more accurate predictions about the effects of antioxidants under biological conditions and the associated implications for human health.

Other future directions for this area of research have been envisioned. Addressing AOX by combining theoretical and experimental efforts is expected to enable the predictions made by computational strategies to be validated.^332^ Investigations that combine computational strategies with experimental results *in vitro* or *in vivo* are, certainly, the ideal approach to conducting this kind of research. A future in which experimental techniques can deal with some of their current major challenges, for example, obtaining detailed mechanistic insights about chemical reactions, or dealing with very short-life species, is easy to foresee. What computational chemistry could possibly offer then? In my opinion, computer-based techniques and computing capabilities are also likely to continue improving. This means that the predictions made by using computational tools would become more reliable. Thus, the virtuous dialog between theory and experiments is probably going to be stronger in the future than it is nowadays. One possible scenario for it, that is already happening, is the discovery of new molecules. *In silico* studies, as a first stage, would contribute to accelerate the process, saving human efforts, resources and, hopefully, even experimentation with animals.

The design of new antioxidants as a potential path to build candidates that fulfill the various requirements of a suitable antioxidant was also proposed.^332^ In this direction, the recently developed protocol “Computer-Assisted Design of Multifunctional Antioxidants, based on chemical properties” (CADMA-Chem)^138^ might be useful. Although, so far, it has been applied to the design of drugs intended for the treatment of OS-related diseases,^333–335^ it can also be used for designing compounds aimed to act as efficient antioxidants.

Machine learning (ML) and artificial intelligence (AI) are emerging as promising tools to advance the research of AOX. Some progress is already evident. The contributions of these techniques to drug design and medicinal chemistry are growing. Deep learning (DL) has been particularly successful for predicting “quantitative structure–activity relationships” (QSARs), ADME (“absorption, distribution, metabolism, and excretion”) properties, and toxicity, as well as for virtual screening and drug repositioning.^336^ Some advances related to using ML-based strategies for identifying antioxidants^337^ and the potential of DL to overcome current challenges associated with modeling PLI using docking simulations^338^ have been recently reviewed.

Artificial neural networks (ANN) were constructed to accelerate VTST calculations^339^ and to investigate the role of functional groups and their positions in the H transfer process involved in the AOX of flavonoids.^340^ ML models trained with conceptual DFT descriptors calculated at GFN1-xTB and GFN2-xTB levels of theory were used to assess the FRS activity of 202 polyphenols towards DPPH.^341^ Twelve peptides meant to be antioxidants were predicted using a DL classification model. After further evaluation using DFT methods, six were synthesized and found to be active in the DPPH assay, while three showed significant affinity for the Keap1 protein.^342^ ML has also been used to reduce the number of false positives arising from docking screenings.^343,344^

Employing random forest (RF) and ANN methods, based on QSAR, the AOX of Se-NSAIDs (“nonsteroidal anti-inflammatory drugs”) derivatives was investigated. The used QSAR-ML approach was proposed as efficient for the intended purpose.^345^ ML was used to predict the influence of the C-chain length, functional groups and their site position on the solubility and BDE of *p*-phenylenediamine (PPD) derivatives. Molecular simulations confirmed the predictions.^346^ An ML-RF model was found to reproduce experimental results for the concentration of three PPDs, and 2-anilino-5-[(4-methylpentan-2yl)amino]cyclohexa-2,5-diene-1,4-dione, in human urine samples.^347^ This finding suggests that ML-RF models might allow overcoming one of the still unsolved challenges involved in computational calculations, including concentration when estimating AOX. A wide range of ML approaches were tested to predict AOX for 130 tripeptides with known activity.^348^ The best one, involving RF for feature selection, was proven to correlate with experimental data, using six of the investigated peptides.

After reviewing some AI-based docking techniques, their potential “to reshape the landscape of drug discovery” was foreseen, and the possibility of enhancing their accuracy by incorporating experimental data into the training was proposed.^349^ The importance of having enough reliable data to ensure the performance of AI-ML models has been highlighted by other authors.^350^ This is key to provide statistically significant predictions from these novel approaches. The limited amount of data available in some research fields is recognized as a drawback of using, for example, DL approaches.^270^ Accurate calculated data are an alternative to time-consuming and expensive experiments. The main problem with high-accuracy calculations is their elevated computational demands. Thus, another area of research that may be expanding shortly is to find less demanding approaches that retain the accuracy of the more time-consuming and resource-demanding ones. For example, it has been demonstrated that PS-ACP methods can act as intermediaries between high-level calculations and the development of DL models.^144^ BDEs, barriers and energies of reaction were calculated within the chemical accuracy and in shorter times than DFT approaches conventionally used for training DL models.

## 5. Concluding remarks

In Halliwell's words, an antioxidant is “any substance that delays, prevents, or removes oxidative damage to a target molecule”.^12^ Such protection against OS is complex and may occur through diverse processes, including chemical and enzymatic pathways. Antioxidants are more than free radical scavengers. They can also act as inhibitors of ˙OH production *via* Fenton-like reactions by chelating redox metals, repair oxidatively damaged biomolecules, and modulate the antioxidant/oxidant enzymatic system. Some of the main mechanisms involved in chemical pathways are formal hydrogen atom transfer (*f*-HAT), single electron transfer (SET), sequential proton lost electron transfer (SPLET), and coupled-deprotonation-chelation mechanism (CDCM). The enzymatic-related protection of antioxidants, on the other hand, includes downregulating or upregulating oxidant and antioxidant enzymes, respectively.

A wide variety of computational approaches can be applied to study antioxidant activity (AOX). Chemical pathways can be evaluated by reactivity descriptors such as bond dissociation energies, ionization energies, redox potentials, and indexes derived from conceptual DFT. Some strategies to investigate these pathways are: (i) thermochemistry, using, for example, Gibbs free energies of reaction, and (ii) kinetics, based on reaction barriers, rate constants, or activation energies. Enzymatic pathways can be investigated by exploring ligand–receptor interactions between candidates and proteins known for their role in the antioxidant/oxidant balance. These studies are frequently carried out using docking methodologies or more computationally demanding calculations, such as molecular dynamics. They can also be combined to gain a more comprehensive understanding of this activity.

Some key aspects to consider when evaluating antioxidant candidates using computational tools are:

(a) There are diverse ways of action and reaction mechanisms that might contribute to AOX.

(b) Solvent and pH of aqueous solution influence chemical reactivity.

(c) Tunneling effects might be significant in certain pathways, such as *f*-HAT.

(d) Free radical scavenging (FRS) processes not only depend on the intrinsic reactivity of the candidate but also on the reactivity of the reacting radical.

(e) The products yielded by FRS should not react with O_2_ or biomolecules. Otherwise, they may contribute to propagate oxidation.

(f) The candidate, the products formed after it reacts with free radicals, and the corresponding metabolites should not be toxic.

(g) Since in living systems biomolecules are in larger concentrations than exogenous antioxidants, the latter should react faster with free radicals to prevent OS.

(h) It is essential to use references or thresholds to put the calculated data into perspective.

The main challenge of using computational tools to predict AOX and establishing trends on this activity arises from the chemical complexity of the processes involving these substances in biological environments. Thus, conclusions should be cautiously drawn from analyzing a limited aspect of the antioxidants' chemistry. Future directions in this area of research may include finding a way to combine results derived from different routes of AOX into a single value, designing new antioxidant candidates, considering the full complexity of the AOX-associated chemistry, increasing combined computational–experimental investigations, and incorporating machine learning and artificial intelligence into this line of research.

Computational tools have been demonstrated to provide valuable molecular insights into AOX chemistry. It is likely that their contributions to the field will continue to enhance the knowledge about antioxidants and their health benefits and that the accuracy of their predictions will increase through the addition of new methodologies to the currently used ones.

## Author contributions

AG: conceptualization, data curation, formal analysis, investigation, methodology, supervision, validation, visualization, writing – original draft and writing – review & editing.

## Conflicts of interest

There are no conflicts to declare.

## Data Availability

No primary research results, software or code have been included and no new data were generated or analysed as part of this review.
